# From individual to interprofessional: characteristics of assessment tasks to assess interprofessional collaboration in healthcare education

**DOI:** 10.1080/13561820.2024.2381058

**Published:** 2024-08-02

**Authors:** Hester Wilhelmina Henrica Smeets, Laurie E. C. Delnoij, Dominique M. A. Sluijsmans, Albine Moser, Jeroen J. G. van Merriënboer

**Affiliations:** a Research Centre for Autonomy and Participation, Zuyd University of Applied Sciences, Heerlen, The Netherlands; b School of Health Professions Education, Maastricht University, Maastricht, The Netherlands; c Department of Educational Research & Development, School of Business and Economics, Maastricht University, Maastricht, The Netherlands; d Research Centre Urban Talent, Rotterdam University of Applied Sciences, Rotterdam, The Netherlands; e Department of Family Medicine, Maastricht University, Maastricht, The Netherlands

**Keywords:** Collaborative learning, collaborative practice, healthcare education, interprofessional assessment, interprofessional education, team-based assessment

## Abstract

To develop independent healthcare professionals able to collaborate in interprofessional teams, health professions education aims to support students in transitioning from an individual perspective to interprofessional collaboration. The five elements that yield the conditions for effective interprofessional collaboration are: (1) positive interdependence, (2) individual accountability, (3) promotive interaction, (4) interpersonal skills, and (5) reflection on team processes. The aim of the current study is to gain insights into how to design tasks to assess a student team as a whole on their interprofessional collaboration. This was a pilot study using a qualitative design to evaluate an interprofessional assessment task. Four interprofessional student teams, comprising physiotherapy, occupational therapy, arts therapy and nursing students (*N* = 13), completed this task and five assessors used a rubric to assess video recordings of the teams’ task completion, and then participated in a group interview. The completed rubrics and the interview transcript were analyzed using content analysis. Findings showed that the combination of individual preparation, an interprofessional team meeting resulting in care agreements and team reflection was a strength of the assessment task, enabling the task to elicit sufficient promotive interaction between students. Areas for improvement of the assessment task were however, due to a lack of interdependence, the care agreements which now proved to be the sum of students’ intraprofessional ideas rather than an interprofessional integration of agreements. Additionally, assessors suggested that a series of varying assessment tasks is required to draw conclusions about students’ interprofessional competence.

## Introduction

Improved interprofessional (IP) collaboration is considered a key strategy for healthcare reform (Bachynsky, 2020; Bosch & Mansell, 2015). There is emerging evidence that when IP healthcare teams practice collaboratively, they can enhance the delivery of person-centered care and lead to improved patient and health systems outcomes (Reeves et al., 2016). IP collaboration is often operationalized using competencies, such as communication, shared decision-making, conflict resolution, and reflection (Interprofessional Education Collaborative, 2016). The adoption of individually framed competencies assumes that if each healthcare professional demonstrates IP competencies, eventually their IP team collaboration will be optimal (McLaney et al., 2022). However, the individual focus causes tension in IP collaboration, as excellent patient care arises not from the efforts of separate individuals, but from the collaboration and interaction among team members. More specifically, delivery of optimal care within an effective IP team is based on the collective efforts of team members and is better accomplished through shared responsibilities, interactive planning and collaborative decision-making (Langlois et al., 2020). These collective efforts of the team as a whole can be seen as a team’s IP collaboration, or a team’s approach of working together to attain group goals that cannot be obtained by working alone or competitively (inspired by Johnson & Johnson, 2002). Therefore, healthcare may benefit from focusing on teams who use IP collaboration, rather than focus on the individual professionals acquiring IP competencies.

Health professions education addressing interprofessional interaction needs to educate students about adopting an IP collaboration approach. Educating students in IP collaboration requires processes that must be developed to determine whether student teams are able to work interprofessionally as expected at the start of their healthcare professional program. Initially, more insight is required into the construct of IP collaboration. Thinking and working interprofessionally calls for different strategies than doing so monoprofessionally. Then assessment can be designed that is aligned with that construct. Though we have seen many developments in the field of IP education, for example, regarding simulation-based learning, or e-learning (El-Awaisi & Waller, 2023), little is known about how to design assessments for assessing this construct of IP collaboration, and how to incorporate assessment of IP collaboration in traditional individual assessment models (Boud & Bearman, 2022; Smeets et al., 2021). Authentic assessment of IP collaboration within student teams poses significant challenges in the educational context, primarily because students ultimately graduate on an individual basis. Consequently, IP assessment requires a constant balancing act between assessment of the collaborative efforts of the team in an authentic manner and assessing individual contributions, which may be less authentic to accurately reflect each student’s performance (Smeets et al., 2023). The aim of the current study is to gain more insights into how to design an assessment task to assess a student team as a whole on their IP collaboration.

Though little is known about the assessment construct of IP collaboration, much research has been conducted regarding the theory of cooperative learning strategies (Johnson & Johnson, 2002). Cooperative learning strategies are especially effective for teaching teamwork skills (D’Eon, 2005). Inspired by Johnson and Johnson’s cooperation strategy, we have defined an approach to IP collaboration to integrate these principles into IP education and assessment. Based on this theory, five elements are essential for IP collaboration that, if followed through, will yield the conditions for effective IP collaboration in an IP team (see Table 1, based primarily on Johnson and Johnson (2002)): (1) positive interdependence, (2) individual accountability, (3) promotive interaction, (4) interpersonal skills, and (5) reflection on team processes.

**Table 1.** Five elements of interprofessional (IP) collaboration.

Element of IP collaboration	Definition
Positive interdependence	Positive interdependence means that students in an IP team strive together to reach a common goal by sharing resources, adopting roles that complement each other, and celebrating their successes together (D’Eon, 2005).
Individual accountability	Individual accountability entails that each team member in the IP team has a personal responsibility for their own professional share of the work that aids the interprofessional work of the team (Johnson & Johnson, 2002).
Promotive interaction	Promotive interaction means close, usually synchronous, purposeful activity such as discussion, debate and joint decision-making where members help each other to succeed (Johnson & Johnson, 2002).
Interpersonal skills	Interpersonal and small group skills are required for contributing to the success of a cooperative effort. To coordinate efforts to achieve mutual goals students must (1) get to know, respect, and trust each other, (2) communicate accurately and unambiguously, (3) accept and support each other, and (4) resolve conflicts constructively (Johnson & Johnson, 2002).
Reflection on team processes	Reflection on team processes entails that students reflect on the actions (both group and individual) that contribute (or not) to the effectiveness of the IP process and deciding what to do or not do about it. Here students think about how the group functioned and what might make it work better, perhaps in light of explicit teaching of a social/relational skill (D’Eon, 2005).

An assessment task for assessing IP collaboration would have two design considerations: (1) it should be designed so that it elicits information about the use of the five elements of IP collaboration, and (2) it should enable assessors to draw valid conclusions about the extent to which student teams are able to use these five elements. Often, such an assessment task is aimed at one particular aspect of IP collaboration, for example, attitude or knowledge, or at the individual student instead of the team of students (Boud & Bearman, 2022; Rogers et al., 2017; Smeets et al., 2021). Previous research demonstrated that students still often use individual strategies in IP assessment (Smeets et al., 2023). Using individual assessments in IP education presents a unique difficulty, because IP performance depends on the context, the task, and the team of students (Boud & Bearman, 2022). For example, individual students may excel when assessed separately on their teamwork knowledge and skills, but perform poorly when truly working together as a team. Currently, assessments focus on individual, measurable outcomes, which contradicts the social aspect of learning (Boud & Bearman, 2022). In IP assessments, the student team’s success may depend on other factors than every student’s knowledge and skills, such as the team dynamics or the applicability of the client cases to their profession. Boud and Bearman (2022) contend that any individual assessment of collaboration is inherently somewhat unfair due to the dependency context, making it inadequate to base decisions about students’ individual IP competence on an assessment task. In the IP assessment literature, little is known about assessing a team’s IP collaboration instead of individual student’s competencies. To gain more insight into the construct of IP collaboration, and into designing IP assessment tasks aimed at assessing IP collaboration of a student team as a whole, the following research question was formulated:

What are the characteristics of a task that can be used to assess the interprofessional student team as a whole on their interprofessional collaboration?

## Method

### Design and setting

This study was part of a larger design-based research project (Smeets et al., 2021, 2022, 2023). The aim of this larger project was to develop design guidelines for the assessment of interprofessional competencies in higher healthcare education. Previous studies in this project regarded a scoping review to understand the state of the art in IP assessment practices, a consensus study to develop design guidelines for IP assessment, and a validation study to evaluate a previous version of an IP assessment task. In the current study, an assessment procedure for second-year students was redesigned based on the results of the previous studies (Smeets et al., 2022, 2023). Student teams completed a prototype assessment task including individual preparation, interprofessional team meeting, the creation of IP care agreements, and team reflection. The main data in this study were collected through assessors assessing videos of student teams performing the IP assessment task, and a subsequent group interview among those assessors.

This study was carried out at two Dutch Universities of Applied Sciences, at the undergraduate, second-year level in the healthcare domain. These two universities were similar regarding their monoprofessional education in years 1 and 2, and at both universities, students came into contact with IP education, but did not have elaborate experience in this field. Students participated in an assessment procedure that was not part of their educational program. The context of the assessment procedure was made as authentic as possible in an educational context, for example, by administering the assessment in a classroom at their university, and by excluding teacher or assessor presence in the room to stimulate interaction among the students.

### Participants

#### Students

A total of 13 students participated in this study, following the educational programs physiotherapy (*n* = 4), occupational therapy (*n* = 4), nursing (*n* = 3), and art therapy (*n* = 2). The inclusion criterion for students was that they were in the second year of their educational program. An exclusion criterion was if they had participated in a previous study from this research project. Also, students were excluded if we had received sufficient registrations from a particular profession to ensure an equal distribution of professions in the teams. Students were convenience sampled, in that we approached them to participate via an electronic learning environment. We invited students to send us an e-mail, if they were interested and willing to participate. Students received financial compensation for their participation. Four student teams (*N* = 13 students) from two Dutch universities participated in this study. See Table 2 for the team constellations. Team Red (*n* = 3) and Green (*n* = 4) were from Zuyd University of Applied Sciences, and team Blue (*n* = 3) and Purple (*n* = 3) from Arnhem Nijmegen University of Applied Sciences. Each team consisted of one student per profession; for example, Team Red consisted of one physiotherapy student, one occupational therapy student and one arts therapy student.

**Table 2.** Constellations of the four participating student teams.

Student team	Professions represented
Team Red (*n* = 3)	1 Physiotherapy student 1 Occupational therapy student 1 Arts therapy student
Team Green (*n* = 4)	1 Nursing student 1 Physiotherapy student 1 Occupational therapy student 1 Arts therapy student
Team Blue (*n* = 3)	1 Nursing student 1 Physiotherapy student 1 Occupational therapy student
Team Purple (*n* = 3)	1 Nursing student 1 Physiotherapy student 1 Occupational therapy student

#### Assessors

Five assessors participated in this study, from four different higher education institutions. Inclusion criteria were that the assessors had expertise in IP education and/or assessment, for example, experience as module coordinator of an IP course. An exclusion criterion was if they had participated in this research project’s previous study where a previous version of the assessment task was evaluated (Smeets et al., 2023). Assessors were sampled purposively via e-mail or phone. Assessors received financial compensation for their participation. Table 3 describes the characteristics of participating assessors.

**Table 3.** Assessor characteristics.

Assessor	Professional background	Current position	Years in (higher) education	Assessment expertise	IP expertise
A	Psychology	Assistant professor in assessment	13	Research on assessment, advisor in assessment	-
B	Nursing	Senior lecturer	35	University Examination Qualification	Developed IP education program including assessment
C	Educational sciences	Manager in educational development and policy	17	Assessment coordinator, research on programmatic assessment, assessment policy	-
D	Physiotherapy & educational sciences	Senior lecturer	28	Educational development including assessment	Development of IP competency framework
E	Pedagogy	Lecturer pedagogical management Child and Education	38	Developed many assessments in vocational and higher education	Participated in IP education project, co-developed IP reflection tool

### Materials

#### Assessment task

In an iterative process, we designed a prototype assessment task. First, an initial prototype, based on literature and previous exploratory studies (Smeets et al., 2021, 2022, 2023), and second, we asked international experts about their vision on crucial elements for IP assessment. The prototype IP assessment task consisted of several consecutive activities, namely, individual preparation, an IP team meeting, and a reflection. Table 4 provides an overview of the prototype assessment task components. For every component of the assessment task, a link is made to the aspect of IP collaboration, and the implications of that aspect of IP collaboration for the design. All assessment materials were originally written in Dutch and were translated to English for dissemination purposes.

**Table 4.** Design features of the prototypical IP assessment task.

Components of the IP assessment task	Design feature based on an element of IP collaboration
Individual preparation	Individual accountability The task includes individual preparation that leads to all students being responsible for their individual share.
IP Team meeting	Positive interdependence The task is aimed at achieving a team goal that calls for contribution by all participating professions. The task includes client cases that call for input from all professions. The task is aimed at assessing a team goal, such as a mutual product, or team processes. Promotive interaction The task includes a purposeful, face-to-face discussion between the students involved in which students help each other to succeed. The task does not include a teacher or assessor, to stimulate independence and interaction between the students. Interpersonal skills The task includes assessment criteria aimed at assessing interpersonal skills (see rubrics)
IP reflection	Reflection on team processes The task includes evaluation of team processes and individual contributions to the team processes.

##### Individual preparation

In IP teams, it is expected that each member contributes and is responsible for their share of the work. To assure this individual accountability, we included an individual preparatory task. Students prepared three or four client cases using a preparation form (Supplemental material 1) summarizing the cases, interventions they recommended from their professional perspective, and their questions for the team members with other professional backgrounds. Each client case had a preponderant focus on one profession, but all cases called for input from multiple professions (Supplemental material 2). Which and how many cases were prepared and discussed corresponded with the professions of the students participating in the team. For example, in Team Red (Table 2), the physiotherapy, occupational therapy, and arts therapy cases were discussed.

##### IP team meeting

Purposeful, face-to-face activity is relevant to assure promotive interaction. An IP team meeting was used for this purpose. Student teams discussed the client cases and formulated care agreements for these cases.

##### Reflection on the IP process

Finally, reflection on team processes was assured by including reflection as an explicit part of the IP assessment task. Students were assigned to consider what went well in their IP collaboration and what could have gone better, discussing this within the team.

##### Rubrics

An initial rubric based on the literature on IP collaboration was created to provide assessors with an assessment tool. This initial rubric was discussed with IP experts from the Netherlands and Belgium as well as with former students, reflecting on completeness and clarity. The rubric used to assess the students’ IP performance consisted of 10 criteria aimed at elements of IP collaboration (Table 5).

**Table 5.** Rubric used in the IP assessment task.

*Element of IP collaboration*	Criteria per component of IP assessment task		
**Preparation – individual score**			
	*Fail – The team member handed in incomplete preparation forms without references (e.g., questions for other professions are lacking).* *Pass – The team member handed in complete preparation forms with references.*		
*Individual accountability*	1. The team member handed in complete preparation forms, with an appropriate summary, evidence-based interventions from their own professional standpoint, and questions for the other professions.	*Fail Pass*	*Feedback*
	Feedback criterion	During the team meeting – team feedback	Met / exceeded the criterion
*Positive interdependence*		2. The team members explain their professional role to the other team members in relation to the client cases, such as their responsibilities, standards, guidelines, boundaries of the own profession.	
*Interpersonal skills*		3. The team members use language that can be understood by the other members, and explain profession-specific terminology/jargon.	
*Positive interdependence*		4. The team members actively ask each other about the possible contributions of the different professions.	
*Interpersonal skills*		5. The team members seek clarification from each other in a respectful way, for example, by asking for an explanation when using subject-specific terminology.	
*Positive interdependence*		6. The team members show that they work in a patient-oriented, way, for example, by prompting each other to formulate care agreements.	
*Positive interdependence*		7. The team members show that they are working toward an interprofessional goal by working together to ensure that no one profession dominates the discussion.	
**Formulating care agreements – group score**			
	*1 – IP novice: The team members discuss the care agreements hardly at all, or do not reach agreement on the care agreements*. *2 – IP-developing: The team members discuss different insights with each other, and agree with each other on care agreements*. *3 – IP-competent: The team members discuss different insights with each other, and agree with each other on care agreements, prioritization of care appointments, and time schedule.*		
*Positive interdependence*	8. The team members formulate specific care agreements per case that express interprofessional collaboration.	***1 2 3***	*Feedback*
	Feedback criterion	Reflection – group score	Met / exceeded the criterion
*Reflection on team processes*		9. The team members name new insights they have acquired about the process of interprofessional collaboration.	
*Reflection on team processes*		10. The team members name new insights they have acquired about themselves by participating in the Interprofessional Team Meeting.	

#### Interview guide

The group interview followed the interview guide (Table 6) and was structured according to the different components of the assessment task. Finally, assessors were asked to reflect on the IP assessment task in general, by indicating its strengths and weaknesses. The interview guide was originally written in Dutch and was translated to English for dissemination purposes.

**Table 6.** Interview guide.

Interview questions
**Preparation** How does the individual preparation contribute to eliciting students’ IP behavior? How does the individual preparation contribute to assessing students’ IP competence? What can be improved in the individual preparation and its instructions? What can be improved in the criteria for assessing the individual preparation?
**IP Team meeting & care agreements** How does the IP team meeting contribute to eliciting students’ IP behavior? How does the IP team meeting contribute to assessing students’ IP competence? What can be improved in the IP team meeting and its instructions? What can be improved in the IP care agreements and its instructions? What can be improved in the criteria for assessing the IP team meeting and care agreements?
**Reflection** How does the reflection contribute to eliciting students’ IP behavior? How does the reflection contribute to assessing students’ IP competence? What can be improved in the reflection and its instructions? What can be improved in the criteria for assessing reflection?
**Evaluating students’ IP competencies** How can the current assessment task be used to make a decision about students’ IP competencies? What needs to be changed in the current assessment task, criteria or implementation thereof to be able to decide about students’ IP competence?
**General** What was your general impression of this IP assessment task? ***Strengths***: What characteristics of the task challenged students to demonstrate IP behavior? What characteristics of the task helped you in making a decision about students’ IP competence? ***Weaknesses***: What characteristics of the task did not (yet) challenge students to demonstrate IP behavior? What characteristics of the task meant that you could not (yet) make a decision about students’ IP competence?

### Procedure

#### Phase 1: student teams performing IP task

The participating students received information per e-mail about completing the prototype IP assessment task, consisting of an introductory letter and an instruction manual describing the assessment procedure. After 2 weeks, the students met at the university to complete the IP assessment task in April and May 2023. On the day of the assessment task, students joined with the researcher (HS) who explained what the session would look like. First, students received the client cases to prepare for the IP team meeting and filled in the individual preparation forms for these cases (1.5 hours). Second, students participated in the IP team meeting, discussing the cases and formulating care agreements. The assessment task finished with a team reflection on the collaboration process (approx. 1 hour per team meeting). Students sent their preparation forms together with the care agreements to HS. All student activities were conducted in Dutch, and all student teams were video-recorded during the IP team meeting.

#### Phase 2: assessors scoring students’ performance

The assessors (*N* = 5) received information regarding the research per e-mail, consisting of an introductory letter and the rubric. All assessors participated in an online calibration session (1.5 hours) in July 2023 in which the assessment criteria were explained, and in which they practiced on a pilot case to develop a shared understanding of the criteria. Subsequently, the assessors received links to the videos of the student teams along with the individual preparation work of students in the corresponding team and their final care agreements. Assessors used an online link to complete the rubric to score the IP performance of each team (using Qualtrics). All assessors assessed the same four student teams individually, within 1 week after the calibration session.

#### Phase 3: group interview

The group interview took place 1 week after the calibration session. One of the researchers (HS) used the interview guide to discuss strengths and weaknesses of the IP assessment task. Another researcher (LD) was present as an observer, to take notes, keep track of the time, and ask additional questions. The group interview was held online via MS Teams, and lasted 1.5 hours. The group interview was recorded and transcribed verbatim. All assessor activities were conducted in Dutch.

### Analysis

To analyze the assessors’ feedback on student team performance a deductive thematic analysis was used (Elo & Kyngäs, 2008). An analysis matrix was developed based on the components of the assessment task. Researchers HS and LD independently coded the assessors’ feedback according to those categories.

The transcript of the group interview was read first by HS and LD to become familiar with the data. These data were analyzed using a combination of deductive and inductive content analysis (Elo & Kyngäs, 2008). For deductive coding the same strategy as explained above for analysis of assessors’ feedback was used. The inductive coding process by open coding was initiated with notes and headings made in the text whilst reading. To limit the number of sub-categories, the lists of sub-categories were grouped under higher order headings. In the abstraction phase a general description of the research topic was formulated. One main category was added through this process, namely *Implications for implementation*, being defined as recommendations with the intention of enhancing overall effectiveness regarding IP assessment procedures. The final analysis matrix can be found in Table 7. The top row consists of the components of the IP assessment task and the added main category, Implications for implementation. The sub-categories are shown under each category.

**Table 7.** Analysis matrix.

Individual preparation	IP Team meeting	IP care agreements	Reflection	Implications for implementation
Assessment criteria Profession-specific focus of the individual preparation Indicates necessity of involving other professions	Assessment criteria Patient centeredness Effectiveness of the meeting Explanation of professional roles Shared language IP attitude Addresses mistakes or things that are unclear	Assessment criteria Includes a time path Patient centeredness	Assessment criteria Content reflection Process reflection	Program of assessment
Formative purpose	Interprofessionalism in team meeting	Consensus about IP care agreements	Formative purpose	Scaffolding

### Ethics

All participants gave written, informed consent prior to participation. Participants were ensured of confidentiality and anonymity. Ethical approval was granted by the research ethics committee from the Faculty of Health, Medicine, and Life Sciences, Maastricht University (number FHML-REC/2020/126/Amendment03_21).

### Trustworthiness

Various approaches were adopted to ensure the trustworthiness of this study (Lincoln & Guba, 1985). Investigator and analyzer triangulation, involving collaboration with multiple researchers during the interview and in the analysis were applied, and participant triangulation involving the inclusion of several assessors with different views on IP assessment. Following the interview, HS provided a concise summary for member checking. The use of detailed descriptions will permit readers to make informed judgments about the applicability of our process and findings to their educational settings.

## Results

In this section, we present the results of the student teams’ assessments and the group interview, organized by the components of the assessment task, and including the additional category Implications for implementation.

### Individual preparation

#### Assessment criteria

In the student assessments, reasons for assessors to score a “pass” for individual preparations were that students met the criterion of a structured and complete case summary, and noted specific questions for other professions. Points addressed by feedback were mainly that the case summaries were lacking one of the elements requested in the rubrics, such as the questions, or the references for the proposed interventions students mentioned.

Neatly structured summary and questions, also described resources and guidelines and suggested care agreements.

(Assessor E, assessing Team Red student)

In the group interview, assessors stated that the individual preparation was a key element in the IP assessment task. The preparation should focus on the students’ own professional background in relation to the patient cases, as well as on preparing for working interprofessionally. It might be too complex to have students ask concrete questions for certain professions. A more achievable step might be to indicate professional boundaries related to the case and the necessity of involving others, without formulating specific questions or stating the professions from which this help should come:

… that they say these are elements in the case that I need help with from others, and then they do not have to know which profession that has to be.

(Assessor A, group interview)

#### Formative purpose

In the group interview the assessors reasoned that decent preparation for completing the current assessment task did not necessarily result in a better team meeting. Student teams in which the individual students scored a “fail” on their individual preparation did not perform worse regarding interprofessionalism in the IP team meeting. Therefore, students do need the preparation as a stepping stone for the IP team meeting, but it does not have to be formally assessed. Preparation should be mandatory otherwise there is the risk that students will skip the preparation.

If someone only writes down keywords that I [as an assessor] don’t understand, but has thought about it and has a good discussion using those keywords, yes, then it’s a prerequisite to enter, but we are not going to assess it.

(Assessor C, group interview)

### IP team meeting – process

#### Assessment criteria

Assessors indicated that face-to-face discussion between students is crucial in IP collaboration. They stated that student teams in the current study did have fruitful discussions about the patient cases, but that these could have been more effective, in terms of time, purposefulness, and costs. Some student teams took (too) much time in discussing the patient cases, which is not in line with these requirements and also not in line with real IP practice.

A first important criterion in the team meeting was patient centeredness. A point of feedback in the student team assessments was that students did not focus sufficiently on the context of the patient, such as their physical capacity or their financial situation. A second important criterion was the explanation of each professional role, which some student teams did, and other teams did not do consistently. Positive feedback was that some student teams explained their own reasoning, and profession-specific tests and interventions in a clear manner. A third key criterion was shared language, which includes avoiding the use of jargon, explaining profession-specific terminology, and explaining abbreviations:

Jargon is used. Terminology mentioned is not responded to, so I have to assume that they understand each other. But that is not checked.

(Assessor C, assessment of Team Blue)

Having the right “IP attitude” was also mentioned in the group interview as an important aspect of IP collaboration. Assessors valued when students demonstrated respect, for instance, by giving each other room to talk, and demonstrating appreciation for each other’s contribution. They stated that such an IP attitude is expressed through openness, curiosity about others, actively searching for other ideas, and active listening. Assessors questioned how interprofessional a team meeting is when one student has an indifferent attitude, which they saw in some of the students:

I watched one video in which the nursing student was so absent, and was busy with a lot of stuff except with the team meeting. Busy with nails, with the phone … It really distracted.

(Assessor B, group interview)

Assessors indicated that it is important that students correct each other, or actively ask questions when something is not clear. In the assessments, assessors wrote that students did not check for each other’s understanding, for instance, when using specific terminology, and that students did not ask each other for explanation. If students did not explain their role, did not use understandable language, or did not demonstrate an IP attitude, their peers did not point this out:

They talked about the Jepsen-test. I don’t know exactly what this is, but other students also don’t know, and they accepted it without asking for clarification.

(Assessor D, group interview)

#### Interprofessionalism in the team meeting

In the group interview, assessors mentioned that this task did not seem to be executed interprofessionally enough. The dialogue between professions was somewhat limited, and students mainly shared ideas from their own perspective. Assessors reasoned that the students’ lack of interprofessionalism could be due to the fact that all students received the same information in the client cases, or to the students’ lack of experience with IP education:

I found the mutual dialogue limited and everyone told their own story from their own vision. It was more multidisciplinary than interprofessional and they agreed very quickly, … , there was too little interdependency.

(Assessor E, group interview)

### IP care agreements – product

#### Assessment criteria

Positive feedback in the assessment of the teams’ care agreements was that the care agreements included prioritization and a time path, and that the care agreements matched the patient’s help-seeking question. In the group interview, assessors reasoned that one of the most relevant aspects in the assessment criteria was patient-centeredness, which did not receive enough weight in the criteria for the care agreements. Assessors thought that the care agreements should include prioritization based on the needs and wishes of the patient, including an argument for that prioritization:

… one or two groups really tried to think “between the lines.” … You can’t assume that the help-seeking question is the right question, and some teams really thought about that, I thought that was very good.

(Assessor A, group interview)

#### Consensus about IP care agreements

The main feedback in the assessments regarding care agreements was that they were mainly constructed as lists of intraprofessional interventions, instead of being constructed through IP decision-making. In the group interview, assessors considered that this could be caused by a lack of interdependency in the assessment task. Students need to be triggered to reach a mutual set of interventions in which they recognize each other’s professions, but also must align the interventions so all patients can be served in the best way possible:

Sometimes, it was a sum of let’s do this and let’s do that, add it all up in the care package and then we’re done. I think that was a pity.

(Assessor E, group interview)

A suggestion to increase interdependency made in the group interview was to instruct students to end up with a maximum of one or two IP care agreements. Another suggestion was to include an obstacle in the task such as the patient’s limited budget to evoke more discussion between students.

When the assignment is to choose the best [care agreement], then it becomes a different discussion, because then you can’t say “we’ll do a little bit of this, and a little bit of that.”

(Assessor C, group interview)

### Reflection on team processes

#### Assessment criteria

The feedback on the reflection noted that the reflection was often superficial, not focusing on process aspects of collaboration, and that students were not self-critical about how they could improve their IP collaboration. In the group interview, assessors saw reflection as an essential element to improve students’ IP behavior. However, they considered the criteria for assessing the reflection to be too vague:

Students check the rubric and indicate that they think they have done all that well - little critical reflection on their IP process.

(Assessor A, assessment of Team Purple)

In the group interview, assessors said that the assessment task should include a reflection about content and about process. The content level could include having student teams explain what they learned about other professions. The process level could include the individual student’s contribution to the team, and the team’s collaboration concerning formulating IP care agreements:

When you aim the assessment at generating an interprofessional outcome, you should let them reflect on that as well, like “How did we eventually do this together, in a collaborative way?”.

(Assessor B, group interview)

#### Formative purpose

Assessors agreed in the group interview that reflection should not be a formal part of assessing IP competencies. Yet, reflection should be used to provide insight into the learning process of the students and provide input for how to improve in a future task. Additionally, they stated that the reflection should not necessarily take place directly after the IP team meeting:

With one team I thought, now you should go home, watch this video, see what you’re doing and see yourself playing with your phone, not actively contributing to the discussion…

(Assessor C, group interview)

### Implications for implementation

#### Assessment program

One strategy suggested by assessors in the group interview to obtain a more longitudinal view of students’ IP competencies was to gather information about IP competence from different sources in an IP portfolio. In this way, an assessor can see growth in students’ development, and reach a decision about achievement of the learning goals. Assessors mentioned that it would be beneficial for the diversity of the assessment tasks to place students in different teams, though this might be at the cost of safety and trust in team reflection:

You have a simulated IP team meeting, we discuss it, you receive feedback, and you put that in an IP portfolio, and after five of those discussions, you have a portfolio with your development and then I can say as a teacher I can give you a pass.

(Assessor C, group interview)

#### Scaffolding in assessment program

Assessors mentioned that the IP experience of second-year students is limited as far as being a starting point for the current assessment task, and that students need more scaffolding. Students require more guidance and practice in IP collaboration, in which involvement and instruction from the teacher gradually decrease. In parallel with the decrease in explicit instruction and support, the assessment task can become more challenging, for example, by increasing the constraints (i.e., less time for the IP team meeting, smaller budget or other restrictions for care agreements):

I think that the level of the students in general was too low for this assessment, because I had a constant sense of they fall back on their own profession and … I think they can be taken a bit more by the hand and then at a later time you let that go more and more.

(Participant C, group interview)

## Discussion

To improve the quality of health professions education, the aim of this study was to gain more insight into how to design an assessment task to assess IP collaboration in student teams. The task components that were evaluated all seem essential for the design of an IP assessment task: individual preparation, a team meeting that stimulates interaction, the creation of a mutual product (e.g., care agreements), and reflection on content and process. It is essential to ensure that the different components contribute to eliciting the elements of a team’s IP collaboration. The four components of the assessment task are discussed in relation to the theory and previous findings, and provided directions for further research and implications for educational practice.

### Individual preparation

Results demonstrate that students’ individual preparation is required to elicit individual accountability. In this preparation, students should focus on their own professional perspective, while also recognizing their professional boundaries. Whilst it is essential to learn about other professions’ backgrounds and perspectives in IP education, it is equally important to gain insight into one’s own profession (van Diggele et al., 2020). However, it seems that a strong professional identity can lead to less IP identity, such that students who need to use their specialized knowledge and skills would probably try to solve a task individually rather than as a team (Shimizu et al., 2022). Taking into account the limited prior experience students in this study had an IP team meeting, an elaborated preparatory task would set them up better for the aspects of positive interdependence and promotive interaction. By formulating the criterion that students should think about the boundaries of their professional roles, it is assumed that this preparation will contribute to interprofessionalism.

Assessors also reasoned that the individual preparation as evaluated with the current criterion was not necessarily related to a better team meeting afterward. It is possible that students in this study did not put as much work into the individual preparation due to limited time, or because the assessment task was not part of the students’ educational programs. Assessors suggested letting go of the pass/fail evaluation of the individual preparation. However, the literature has suggested that this might result in undesirable behavior such as freeriding, and thereby might hinder the actual elicitation of individual accountability (D’Eon, 2005; Meijer et al., 2020). An alternative suggestion might be to focus the assessment of the individual preparation work on the depth of the individual student’s reflection in exploring the boundaries of their own profession rather than on the completeness of their preparation and how they include references. Further research should be conducted on the balance between intraprofessional and interprofessional preparation that leads to effective completion of the IP assessment task.

### IP team meeting

There are two points to discuss regarding the IP team meeting. First, it seems important that the IP assessment task elicits IP discussions about the formulation of client-centered care agreements. The experience of solving problems as part of a healthcare team is an important mechanism for students to develop an understanding of their own roles, the other professions’ perspectives and how to work together to achieve a common goal (Maddock et al., 2023). However, this task did not elicit enough IP behavior in students’ discussions and in addressing mistakes or unclear issues with each other. There could be several possibilities as to what hindered having a genuine IP discussion, such as lack of experience with IP education or IP collaboration, or students being placed in teams whose members were all unfamiliar to each other (van Diggele et al., 2020). The main focus on assessment of the process of IP collaboration rather than the product (i.e., care agreements) could have hindered genuine IP discussion. In line with previous studies, this suggests that the outcome of the IP process should be considered equally as important as the IP process itself (Dijkstra et al., 2016; Smeets et al., 2022).

Second, based on what the assessors said in this study, an IP assessment task should focus on the development of IP competencies instead of merely on one aspect of competence, namely skills. Skills are often domain-specific and deal with whether or not students can perform a task according to the standards. Key to IP competence is the integration of not only skills, but also knowledge and attitude (Baartman & de Bruijn, 2011). Assessors indicated that a key element of IP behavior is also being daring and being able to point something out to each other or to ask questions. Having an IP attitude was also considered key, for example, not looking at mobile phones during a conversation. It is important to use the right terminology in IP theory. It is proposed that for IP assessment, we need to redefine interpersonal skills and supplement them with competencies relevant for IP performance. The proposal is to change the term “interpersonal skills” to “IP competencies.”

### IP care agreements

According to assessors, what potentially hindered positive interdependence in this assessment task was the lack of restrictions in the end product, which led to students making a relatively long lists of intraprofessional points of agreement. Ultimately, in IP education and IP assessment, student teams must demonstrate a professional and an IP identity, since this is essential to interprofessionalism (Polansky et al., 2023). This is learned and practiced through positive interdependence, a key element of IP collaboration (D’Eon, 2005). Assessors made suggestions on how we can incorporate that positive interdependency into an IP assessment task, such as building restrictions into an assessment task where students are only allowed to formulate a certain number of agreements about care in an IP team. What remains underexplored is how we should match this concept of interdependency to students’ level and educational year. The international literature on identity seems to suggest that students first need to have some training in their own profession before coming together to learn in IP teams, so they have developed their professional identity to some degree (Shimizu et al., 2020). Therefore, we cannot expect full positive interdependence from first-year students. In the final year of their educational programs, students are expected to be able to perform a task in which they are fully dependent on other professions (van Diggele et al., 2020). It is advised, however, to initiate interaction among professions at an earlier stage, because social identity theory suggests that the more students identify with their own profession, the less accommodating they will be to other professions (Haslam, 2017). It remains unclear how assessment tasks should be designed to elicit positive interdependence in entire curricula that build up in complexity.

### Reflection

When assessing IP performance, it is necessary to reflect on team processes. Assessment of such reflection should adopt a formative approach, while focusing on the content and process of IP collaboration. The literature has demonstrated that IP reflection requires metacognitive competence regarding (1) oneself, (2) the other professions on the team, and (3) the team itself (Clark, 2009). It is questionable, however, whether second year students have the metacognitive abilities to do this, and not all individual students in IP teams are equally reflective, given their different personalities and professions (Clark, 2009). Since reflection is a complex task for students, it also is important that students receive specific training in this skill, and to take into account that they must share personal thoughts with each other, requiring a certain level of safety and trust.

For practical implications about the design of an IP assessment task resulting from this study, see Box 1.

**Box 1.** Implications for the design of IP assessment tasks.

**Practical implications** Assess a team of students instead of individual students to assess their IP collaboration. Incorporate both IP process and IP product as assessment targets. Focus on the broad area of IP competencies and the integration of knowledge, skills, and attitudes to assess the student team’s IP performance. Create an IP assessment task in which students have the assignment to reach a common goal that cannot be met intraprofessionally. Align IP education with the IP assessment task, and start with simpler tasks with more guidance (scaffolding), and then move on to more challenging ones. Use a programmatic approach to assess and provide feedback to student teams on their IP collaboration in different settings, in different teams, with a different focus per assessment.

### Strengths and limitations

A strength of this study was the level of authenticity of the assessment task in the context of second-year educational programs. An IP team meeting appears to be an authentic assessment task (Smeets et al., 2023), and second-year students are used to having case-based discussions in a classroom setting. However, there are some challenges regarding the authenticity as well. Students who participated in this study had limited IP experience, whereas it is a key aspect of fair assessment that students are offered appropriate IP learning opportunities to practice IP collaboration through various authentic tasks (Rogers et al., 2017; Smeets et al., 2023). In the first years of IP education, the primary focus should be on developing awareness and knowledge from a monoprofessional perspective, such as understanding how one’s profession relates to others in hypothetical scenarios (Van Merriënboer & Kirschner, 2018). Eventually, a transition to more collaborative tasks is necessary to prepare students authentically for healthcare practice. The students who participated in this study were not entirely new to IP practices, yet their initial exposure was predominantly knowledge-oriented rather than action-oriented. This transition necessitates many practice opportunities and ample feedback in a safe setting for both student teams and individual students.

Also, the recording of the assessment task can be regarded as a limitation, as it can lead to students trying to change their behavior because they are being observed (“Hawthorne effect”). However, in an assessment program or series of completions of this assessment task, it can be desirable for students to have recordings of their own team working interprofessionally to reflect on and learn from. In this way, recording an IP assessment task can become an authentic educational situation.

Another strength lies in the diversity of the assessors who participated in this study, who had different backgrounds and differing expertise, and were thus likely to attend to different characteristics of the IP assessment task. In this way, we reached a comprehensive overview of characteristics of an IP assessment task that assesses a team’s IP collaboration.

A limitation of this study was the narrow focus on a specific IP assessment task in two universities in one country, making the conclusions possibly specific to the Dutch context. Indeed, according to guidelines for design-based research and requirements based on modern validity theories, the validity and impact of assessment tasks are heavily context-dependent and should be considered in the specific context for which the task is designed (Cook et al., 2015). However, by modeling the steps in a design-based validation process, this study might still inspire the systematic validation of IP assessment tasks in other educational contexts.

## Conclusion

The combination of individual preparation, IP team meeting resulting in care agreements and team reflection is the strength of the IP assessment task. This task fostered meaningful interaction among students. Nonetheless, the absence of positive interdependence resulted in care agreements that predominantly reflected students’ intraprofessional perspectives, rather than the integration of an IP consensus. A single assessment is also not enough to make a decision about a student team’s IP collaboration. A graduated program of IP assessments is required, with a gradual increase in complexity and a reduction in guidance and support, in which the assessment tasks are designed to elicit increasingly higher levels of positive interdependence between students in an IP team. Further research is needed to find out what an assessment program looks like that both elicits IP collaboration in student teams and enables educators to make decisions about a student team’s IP competence.

## Supplementary Material

Supplemental materials JIPC.docx
