# Supplementary material for: From individual to interprofessional: characteristics of assessment tasks to assess interprofessional collaboration in healthcare education
Source: J Interprof Care. 2024 Aug 2;38(5):907–17. doi: 10.1080/13561820.2024.2381058 (PMC11374113; doi:10.1080/13561820.2024.2381058)
Supplement: Supplemental materials JIPC.docx [file IJIC_A_2381058_SM5587.docx]

**Supplemental material 1 (translated from Dutch to English for dissemination purposes)**

*Preparation form*

| **Part A: Summary of the client’s situation** | | |
| --- | --- | --- |
| *Date of team meeting:*  *Client name:*  *Client background information:*  *Request for help:* | | |
| **Domain** | **Domain applied to client situation (what is already known and what do you still want to know?)** | |
| **Body functions** |  | |
| Feeling healthy  Fitness  No complaints or pain  Sleep  Eating  Sexuality  Fitness  Moving |  |  |
| **Mental well-being** |  | |
| Remembering  Concentrating  Communicating  Being happy  Accepting yourself  Dealing with change  Sense of control |  |  |
| **Meaningfulness** |  | |
| Meaningful life  Appetite for life  Wanting to achieve ideals  Having confidence  Acceptance  Gratitude  Continuing to learn |  |  |
| **Quality of life** |  | |
| Enjoyment  Being happy  Feeling good about yourself  Balance  Feeling safe  Intimacy  How you live  Getting by with your money |  |  |
| **Participation** |  | |
| Social contacts  Being taken seriously  Doing fun things together  Support from others  Belonging  Doing meaningful things  Interest in society |  |  |
| **Daily functioning** |  | |
| Taking care of yourself  Knowing your limits  Knowing about health  Dealing with time  Dealing with money  Being able to work  Being able to ask for help |  |  |
| **Part B: Opportunities for diagnosis and treatment/interventions** | | |
| Write down which of the client’s problems you can help with based on your profession; this can be either diagnostics or therapy. Keep the client's needs in mind. Support your contribution with evidence, such as scientific literature, guidelines and protocols. Write the (internet) source behind any research or interventions you have mentioned.  Next, write down questions you would like to ask the other professions about this case. What questions would you want to ask the speech therapist, for example, or the occupational therapist? How might the other professions help you in understanding the case? Are there issues where there is overlap between what you and another profession can offer help with? How do you deal with efficiency of care (how do you keep care affordable)? | | |
| **Profession** | | **Contribution by profession and questions for the other professions.** |
| **Physiotherapist** | |  |
| **Occupational therapist** | |  |
| **Speech therapist** | |  |
| **Arts therapist** | |  |
| **Nurse** | |  |
| **Other profession(s)** | |  |
| **Part C: Ultimate care agreements for your case** | | |
| *Care agreement 1*  *Care agreement 2*  *…* | | |

*Based on: Institute for Positive Health (2023)*

**Supplemental material 2 (translated from Dutch to English for dissemination purposes)**

*Client cases*

***Nursing case – Mrs. Hendriks***

A 34-year-old client (Mrs. Hendriks) lives with her family (husband and son). Mrs. H had a cerebrovascular accident (CVA) (left hemisphere of the brain) a few days ago and has been home from the hospital for 2 days now. The district nurse made a home visit to see exactly what is needed for rehabilitation. At the hospital, she had immediate thrombolysis. Mrs. H needs to take medication at home, but indicates that in the past (e.g., antibiotic courses) she often forgot to take the medication.

Mrs. H drags her right foot a lot and she is incredibly tired when she tries to walk a little. She cannot stand for long periods at a time. She already worries if she will ever be able to return to her job as a hairdresser, and if she will ever be able to ride a bike since she does not have a driver's license. She quickly gets a heavy feeling in her legs with light exertion. Fine motor skills are impaired, so she has difficulty with self-care. Getting dressed, brushing teeth, washing/combing hair does not go well. Problems are also expected in cooking and housework. The Jebsen test was given: On this it became clear that her right side scored far below the norm: her right hand was twice as slow as her left hand. Mrs. H is right-handed.

Immediately after the CVA, Mrs. could not speak at all and did not understand what was being said to her. Now she still suffers greatly from word-finding problems, and reports difficulty understanding written text. Mrs. H also chokes easily because she has difficulty chewing. The family has financial worries, and is very worried about their wish to have a second child.

**Medication**

- Clopidogrel (platelet aggregation inhibitor) 75 mg 1D1T
- Pantoprazole 1D1T
- Hydrochlorothiazide TEVA 25 mg 1D1T

**Assistance request**

- Mrs. H indicates she wants to "get back to her old self" and be able to care for her child again.

***Arts therapy case – Ms. Vandenberg***

Ms. Vandenberg (age 51) is dealing with a combination of somatoform pain disorder, posttraumatic stress disorder (PTSD), depression and substance abuse (alcohol). Several months ago, a fall landed Ms. V in the hospital with an ugly shoulder fracture. The fracture prevented her from playing sports and working. After a rehabilitation period, she is now back home. She suffers increasingly from gloominess, insomnia and relapses. In addition, there is avoidance. She is more skittish and experiences a lot of stress, anger and intense sadness. She cannot yet go to work and is not allowed to play sports.

Ms. V has previously had successful (PTSD) treatment where the focus was on processing the past. She was also treated for depressive episodes exacerbated by a divorce at the time. Working and being active was her way of coping to deal with problems and her past. Ms. V does not like life now, avoids people and goes nowhere. Traumatic memories come back.

After intake by a general practice mental health worker, recurrent major depression was diagnosed in addition to PTSD. The depression is in the foreground. In the past she had taken an antidepressant, but preferred not to use it again because of the side effects at the time. It was agreed to monitor the symptoms and in case of insufficient effect of possible interventions to still use the pharmacotherapy module. Ms. has a lot of pain throughout her body, for which no clear cause could be found. Ms. V indicated excessive alcohol consumption (average of 15 glasses of alcohol per week). She started lifestyle training aimed at changing her behavior regarding alcohol consumption, but she doesn't see the point of it.

**Request for help:**

- She would like to be less gloomy again, and "participate" as she used to.

***Occupational therapy case –Smith Family***

Mr. (67 years old) and Mrs. (66 years old) Smith live together in a rented house, in a small cozy neighborhood. They have a small pension. Mr. S was a bicycle mechanic, and after his retirement he did some extra work repairing bicycles. Mrs. S has always earned a little extra by what she calls "cleaning houses". Together they have 3 children, 2 sons and a daughter. Both sons are married. They have 5 grandchildren.

After his second transient ischemic attack (TIA) 2 years ago, Mr. S retired. A year after his retirement, Harm suffered a major cerebrovascular accident (CVA). Not much recovery is in sight. He has loss of strength and spasms of his right arm and drop foot in his right leg. This is his dominant side. Mr. S also can no longer brush his teeth properly (due to decreased strength in his arm). He has inflammation of the gums and lower jaw (periodontitis), which has caused his teeth to become loose here and there. Due to medication, he often has a dry mouth and difficulty swallowing. Mr. S has type II diabetes mellitus (age-related diabetes) and his blood pressure is RR 120/70, pulse 70/min.

Mr. S sometimes falls. He has disease awareness, but not much insight. He has changed since the CVA; he is rigid, can't handle change well and has become grumpy. Mr. S is a talkative man who now suffers from word-finding problems, which he is embarrassed about. His language comprehension is impaired, which is not so noticeable in simple communication. But he can't do much anymore with long sentences, complex language and word jokes.

Mr. S recently had a fall, which greatly shocked him and his wife. Mr. S himself would prefer that everything stay as it was. He was startled by the fall, but does not think there is a problem. Yes, he does know that he gets dizzy sometimes, but that always goes away on its own. He also doesn't expect that he could fall again.

Mrs. S is overweight, and she suffers from worn-out knees. She doesn't dare leave her husband alone anymore; she is afraid he will have another CVA. Or that he will snack too much and his sugars will go haywire. What she does not know is that Mr. S does not eat when he is home alone. He simply forgets to eat or doesn't feel like preparing anything for himself.

The house: small upstairs bathroom, no modifications. It can't have a stair elevator. The housing association suggests senior housing, but that means moving to another neighborhood. And it's more expensive. The cost of new furnishings is also an obstacle.

**Request for help:**

- The couple would like to stay together as long as possible. But they don't know how to proceed.

*(Based on: N. Looman et al., IP case used by Radboud University & University of Arnhem / Nijmegen)*

***Physiotherapy case – Mr. Janssen***

A 74-year-old patient Mr. Janssen, known to have COPD, is still living independently, but indicates himself that things are becoming increasingly difficult at home because of physical complaints. During the visit to the doctor, Mr. J indicates that he is not doing so well. He suffers a lot from coughing. As a result, he does not sleep well, which makes him feel even worse, and he is tired during the day. Mr. J frequently sags through his legs due to fatigue. He says his condition worsened recently, but still tries to seek social contacts.

Increased risk of falling is present; Mr. J has not fallen in the past 12 months. He wears glasses and uses hearing aids; nevertheless, he continues to complain of poor hearing.

Mr. J is not fully independent regarding activities of daily living. For some time, help with putting on and removing compression stockings has been coming twice daily. There is a restaurant in Mr. J’s building where he goes to eat daily; he no longer cooks himself. Once a week a maid comes for major cleaning of the apartment. Mr. J tries to keep the small things clean himself. His action radius is limited; he walks in the house with a walker and can only walk a maximum of 200 meters with a walker outside the house. He would like to be able to move around more independently outside the home, for example, to be able to go to the store by himself again.

Mr. J needs social contact. However, his poor hearing also means that contact is not easy. He indicates that he is not yet completely satisfied with his day; he needs to see people, he has little to do and therefore often watches TV.

**Medical history**

- 1998 Angina pectoris
- 2006 Atrial fibrillation
- 2011 Diabetes Mellitus type 2
- 2012 Tachy-brady syndrome for which pacemaker
- 2012 COPD
- 2015 Hypertension
- 2015 Hypercholesterolemia
- 2019 Cognitive impairment (Mild Cognitive Impairment)

**Medication**

- Salbutamol aerosol 4 puffs
- Colecalciferol capsule 5600IE
- Apixaban 2.5mg 1D1T
- Simvastatin 40mg 1D1T
- Amiodarone 200mg 1D1T
- Beclomethasone/formoterol aerosol 2 puffs
- Macrogol 1-3D1 sachets
- Pantoprazole 40 mg 2D1T
- Prednisolone 30mg 1D1T (7-day course)

**Request for help**

- To be able to function independently at home for as long as possible.
